# Bioactive Secondary Metabolites from *Harposporium anguillulae* Against *Meloidogyne incognita*

**DOI:** 10.3390/microorganisms12122585

**Published:** 2024-12-13

**Authors:** Dong Li, Ling-Feng Bao, Hong-Mei Lei, Guang-Ke Zhang, Guo-Hong Li, Pei-Ji Zhao

**Affiliations:** 1 State Key Laboratory for Conservation and Utilization of Bio-Resources in Yunnan, School of Life Sciences, Yunnan University, Kunming 650091, China; ddll1998@outlook.com (D.L.); 18487146962@163.com (H.-M.L.); 15138938042@163.com (G.-K.Z.); ligh@ynu.edu.cn (G.-H.L.); 2 Institute of Tropical Eco-Agricultural Sciences of Yunnan Academy of Agricultural Sciences, Kunming 650091, China; blf1128@yaas.org.cn

**Keywords:** endoparasitic fungus, *Meloidogyne incognita*, cyclic peptide, nematicidal activity, chemotactic activity

## Abstract

Root-knot nematodes (RKNs) are pathogens that endanger a wide range of crops and cause serious global agricultural losses. In this study, we investigated metabolites of the endoparasitic fungus *Harposporium anguillulae* YMF1.01751, with the expectation of discovering valuable *Meloidogyne incognita* biocontrol compounds. Based on results obtained by a liquid chromatograph coupled to a mass spectrometer (LC-MS) of crude extracts under five culture conditions and their nematicidal activity against *M. incognita*, corn meal agar (CMA) medium was determined as the scale-up fermentation medium. Twelve metabolites (**1**–**12**) were isolated from the fermentation products, and compound **1** was identified to be a new cyclic tetrapeptide. The activity assay results showed that phenylacetic acid (**11**) had good nematicidal activity at 400 μg/mL, and the mortalities of *M. incognita* were 89.76% and 96.05% at 12 and 24 h, respectively, while the mortality of canthin-6-one (**2**) against *M. incognita* was 44.26% at 72 h. In addition, the results of chemotaxis activity showed that 1-(1*H*-indol-3-yl)ethanone (**10**) possessed attraction activity towards *M. incognita*. At the tested concentrations, cyclo-(Arg-Pro) (**4**) and cyclo-(Val-Ile) (**7**) showed an avoidant response to *M. incognita*. This study provides insight into the nematode-active compounds of *H. anguillulae* origin and offers new opportunities for the development of RKN biocontrol products.

## 1. Introduction

Root-knot nematodes (RKNs) are an important pathogen group that parasitizes and proliferates on a wide range of crops, causing significant damage to agricultural production [1,2]. The control of RKNs remains a worldwide challenge, and various methods, including agricultural, physical, chemical, and biological controls, have been applied to prevent RKNs infestations [3,4]. Among them, biological control is an effective method due to its long-term, stable, relatively safe, and environmentally friendly characteristics [5,6].

Endoparasitic fungi produce spores, some of which are swallowed by nematodes and cause them death through spore germination [7,8]. *Harposporium anguillulae* is a typical species of endoparasitic fungi [9]. During infection, the spores of *H. anguillulae* are swallowed by the nematode and then attach to its mouth, intestine, or throat. When the spores germinate into hyphae, they penetrate the nematode’s body wall and consume it for nutrients, ultimately killing them [10]. Recently, some reports indicated that metabolites play an important role in fungal infections of nematodes [11,12]. Our previous work reported that a new furan harposporin A and a known aureonitol were identified from *Harposporium* sp. YMF1.01735 [13]. *H. anguillulae* YMF1.01751 yielded seven metabolites, one of which was a newly identified polyketone, with some showing weak nematicidal activity or chemotactic effects toward nematodes [14], indicating that metabolites of the strain may play a role in parasitizing nematodes. In order to obtain more active metabolites, we further investigated the metabolites of *H. anguillulae* YMF1.01751 through screening culture conditions to establish a chemical framework for understanding the biological mechanisms of *H. anguillulae* YMF1.01751 against *M. incognita*.

## 2. Materials and Methods

### 2.1. H. anguillulae Strain and Culture

*H. anguillulae* YMF1.01751 was isolated from soil and deposited in the Microbial Library’s Germplasm Bank of Wild Species from Southwest China, Yunnan University, Kunming, China. The strain was incubated on PDA medium plates at 28 °C for 5 d in the dark (The medium was made from 200 g of potato, 20 g of glucose, 20 g of agar, and 1 L of water. The peeled potatoes were weighed and boiled for 30 min, and the potato juice was filtered through gauze until a volume of 1 L was obtained. Then, 20 g of glucose and 20 g of agar were added to the potato juice). Then, the colonies were cut into small pieces with a sterile scalpel and transferred to an Erlenmeyer flask filled with PDB (PDA media without agar). The conidia were fully shaken in a shaker for 40 min and then filtered to obtain the conidial liquid for follow-up experiments.

### 2.2. Screening of Culture Conditions

Five solid types of media (PDA, PDYA, WB, OAT, and CMA) were prepared in 500 mL volumes as follows: PDA; PDYA (200 g of potato, 10 g of glucose, 5 g of tryptone, 4 g of yeast extract, 1 g of (NH_4_)_2_SO_4_, 2 g of KH_2_PO_4_, 1.5 g of MgSO_4_, 0.1 g of CaCl_2_, 0.01 g of FeSO_4_, 20 g of agar, and 1 L of water); WB (50 g of wheat bran, 10 g of glucose, 5 g of tryptone, 4 g of yeast extract, 1 g of (NH_4_)_2_SO_4_, 2 g of KH_2_PO_4_, 1.5 g of MgSO_4_, 0.1 g of CaCl_2_, 0.01 g of FeSO_4_, 20 g of agar, and 1 L of water); OAT (50 g of oat, 10 g of glucose, 5 g of tryptone, 4 g of yeast extract, 1 g of (NH_4_)_2_SO_4_, 2 g of KH_2_PO_4_, 1.5 g of MgSO_4_, 0.1 g of CaCl_2_, 0.01 g of FeSO_4_, 20 g of agar, and 1 L of water); CMA (50 g of maize, 10 g of glucose, 5 g of tryptone, 4 g of yeast extract, 1 g of (NH_4_)_2_SO_4_, 2 g of KH_2_PO_4_, 1.5 g of MgSO_4_, 0.1 g of CaCl_2_, 0.01 g of FeSO_4_, 20 g of agar, and 1 L of water). These media were sterilized at 121 °C for 30 min and then placed onto 25 plates (90 mm). The conidial suspension (500 μL) of *H. anguillulae* YMF1.01751 was inoculated on each plate and incubated at 28 °C for 21 days in the dark. The solid culture system, including the media and mycelia, was extracted exhaustively three times using ethyl acetate/methanol/acetic acid (80:15:5, *v*/*v*/*v*), and these extracts were named PDA21, PDYA21, WB21, OAT21, and CMA21. All extracts were detected using LC-MS.

### 2.3. LC-MS Detection and Metabolomic Data Analysis

LC-MS data were obtained using a Dionex UltiMate 3000 LC system coupled with a Q-Exactive Orbitrap mass spectrometer (San Jose, CA, USA) (Thermo, Bremen, Germany). All samples were separated on an XTerra^®^ MS C18 (150 mm × 3.9 mm, Waters, Milford, MA, USA) with a particle size of 5 µm at an LC flow rate of 1.0 mL/min, a flow split ratio of 0.3 directed to the ionization source, and a column temperature of 40 °C. Mobile phase A was 0.1% formic acid in water, and mobile phase B was 0.1% formic acid in methanol. The 35 min gradient using for the positive ESI mode was set as follows: 0–5 min, 10% solvent B; 5–23 min, 10–99% solvent B; 23–28 min, 99% solvent B; and 28–35 min, 10% solvent B. The injection volume was 5 μL, and each sample was injected in triplicate. Untargeted metabolomics data were analyzed using Compound Discoverer 3.3 (Thermo Fisher Scientific, Waltham, MA, USA) following our previous method [15].

### 2.4. Instruments, Extraction, and Isolation

The general experimental instruments, metabolite extraction and isolation, and known compounds’ spectral data are described in the Materials and Methods Section of the Supplementary Materials.

### 2.5. The Nematicidal Activity of Fermentation Products and Compounds

The *M. incognita* used in this study was provided by Dr. Lu CJ [16] and was identified previously as references [17]. The direct contact method [18] was used to assay the nematicidal activity of *H. anguillulae* fermentation extracts against *M. incognita*. First, sterile water containing 1% methanol was used to fully dissolve each of the five extracts (400 μg/mL). Secondly, a water solution containing 200–300 *M. incognita* J2s was added to the 24-hole biochemical test plate (NEST, Wuxi NEST Biotechnology Co. Ltd., Wuxi, China) in the middle of each hole. The solution with 1% methanol and without compounds was used for the blank control group, and each experimental group was designed with 3 parallel assays. Finally, the test plates were incubated at 25 °C. The number of living and deceased nematodes was counted every 12 h, and the death rate of the nematode was calculated.

All isolated compounds were dissolved in methanol. About two hundred *M. incognita* J2s were added to each test and the final concentration of the tested compounds was set at 400 μg/mL. The ddH_2_O, with an equal amount of methanol, was used for the blank control, and avermectin (Macklin, Shanghai, China) was used for the positive control. Nematode mortality was calculated by tracking the total and deceased nematode numbers every 24 h [19]. A stereomicroscope was used to count the number of nematodes at 25 °C. Juveniles were judged deceased if their body was straight without movement despite physical stimulation with a fine needle. The number of survivors and deaths among the root-knot nematodes was counted, and the mortality rate was calculated according to the following formula: Nematode mortality rate (%) = number of dead nematodes/total number of experimental nematodes × 100.

### 2.6. Chemotaxis Assay Against M. incognita

The WA medium (20 g agar, 1 L water) was poured evenly, at a thickness of approximately 1 cm, into a 90 mm Petri dish. After the medium solidified, the central area at the rear of the Petri dish was chosen as the focal point of the circle. Two diameters were drawn around the center, one of which was used as the reference line for adding test samples. A dotted line perpendicular to the reference line was drawn on the left and right sides, 5 mm from the circle’s center, to serve as the sampling area for the test nematode. Then, the sampling reference lines on the left and right sides, 25 mm away from the center of the circle, were marked as S (the experimental group) and C (the blank group), and the sampling area for *M. incognita* J2s was used.

All compounds (**1**–**12**) isolated from *H. anguillulae* YMF1.01751 were dissolved with methanol, and the prepared methanol solution containing the compounds (40, 20, 10, and 5 μg) was added to the area marked S; methanol (with the same volume and no compounds) was added to the area marked C as a blank control. After the solvent dried, an equal volume of aqueous solution containing 200~300 *M. incognita* J2s was added to the circle’s center and cultured at 25 °C. The root-knot nematodes in regions S and C were counted at three time points of 1, 2, and 3 h, and the chemotactic index was calculated according to the following formula [20]: Chemotactic index (CI) = (S − C)/(S + C). CI ≥ 0.2 indicates strong attraction; CI ≥ 0.1 indicates weak attraction; CI ≤ −0.1 indicates weak repulsion; CI ≤ −0.2 indicates strong repulsion; and −0.1 < CI < 0.1 indicates random movement.

## 3. Results

### 3.1. Activity Assay and LC-MS Detection of Fermentation Products Under Different Screening Conditions

The nematicidal activity of the extracts from five media types against *M. incognita* was measured. The results showed that at a concentration of 400 μg/mL, the nematicidal activity of the PDYA, CMA, and OAT extracts was significantly enhanced at 24, 36, and 48 h and tended to be stable at 48 h, with mortalities of 76.2%, 47.0%, and 12.5%, respectively. Two way-ANOVA was used for statistical analysis with the software GraphPad Prism 9.5.1 (Figure S1), and the results indicated significant differences of nematicidal activity between three media extracts and control. The WB and PDA extracts had no obvious nematode-killing activity at 400 μg/mL.

**Figure 1:**
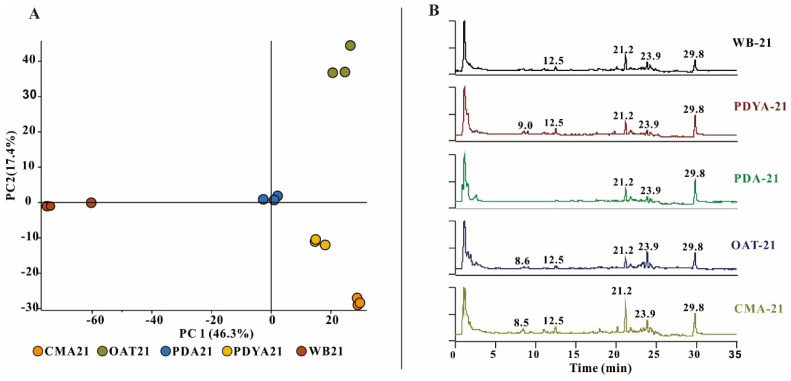
The LC-MS detection of extracts produced by *H. anguillulae* YMF1.01751 in five media. (**A**) Principal component analysis of the metabolites from *H. anguillulae* YMF1.01751 in different media. (**B**) LC-MS profiles of metabolites extracted from *H. anguillulae* YMF1.01751 in total ion chromatography.

To determine whether there were differences in metabolites in the different culture media, the five samples were subjected to untargeted metabolomics LC-MS. Relative quantitative analyses of metabolites may indicate metabolite differences in *H. anguillulae* in different culture media. The metabolomics data for the principal component analysis (PCA) were derived from all metabolites annotated by Compound Discoverer 3.3 after the LC-MS analysis. The results of the PCA showed that the extracts of the three replicates had good aggregation under the same culture conditions. The metabolites of *H. anguillulae* YMF1.01751 varied under different culture conditions (Figure 1A), as analyzed using LC-MS chromatograms. We found that the extracts from CMA had significantly more peaks at 8–13 min and 16–25 min than the other extracts in the total ion chromatogram (Figure 1B). Based on the LC-MS detection and nematicidal activity results, CMA was ultimately selected to ferment the strain *H. anguillulae* YMF1.01751.

### 3.2. Structural Identification of the Compounds

A total of 12 metabolites were isolated and identified from the extracts of *H. anguillulae* YMF 1.01751 on the CMA medium.

Compound **1**: White solid; C_18_H_32_N_4_O_4_; ESI-MS *m*/*z*: 369 [M + H]^+^; ${\left[\alpha \right]}_{D}^{25}$ = −69.2 (c 0.10, MeOH); for ^1^H-NMR (CD_3_OD, 600 MHz) and ^13^C-NMR (CD_3_OD, 150 MHz) data, see Table 1. HR-ESI-MS: 369.2483 ([M + H]^+^, calc. for C_18_H_32_O_4_N_4_, 369.2496). The results of the positive HR-ESI-MS analysis indicated a molecular formula of C_18_H_32_N_4_O_4_ based on the [M + H]^+^ ion peak at *m*/*z* 369.2483 (calc. 369.2496). The ^13^C-NMR and DEPT data (Table 1) revealed four quaternary carbons (*δ_C_* 171.5, 171.1, 169.2, and 171.0), six methines including four nitrogen-substituted methines (*δ_C_* 51.7, 51.9, 60.9, and 54.6), two methylene groups, and six methyl groups. According to the reference data, compound **1** may be a cyclic tetrapeptide [21,22].

**Figure 2:**
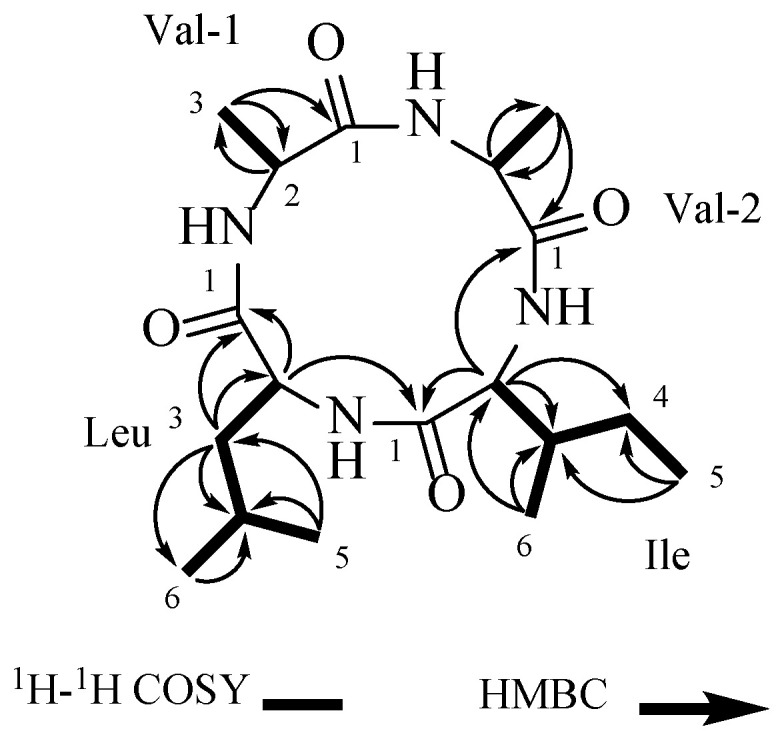
The COSY and key HMBC correlations of **1**.

The connections of the amino acid residues in the structure of **1** were established using key correlations from the 2D-NMR data (Table 1): Val-1-H-3 (*δ_H_* 1.43) has an HMBC remote correlation with Val-1-C-1 (*δ_C_* 171.5) and Val-1-C-2 (*δ_C_* 51.7), and Val-1-H-2 (*δ_H_* 4.03) and Val-1-H-3 (*δ_H_* 1.43) showed COSY correlations, establishing one valine residue (Val-1). Val-2-H-3 (*δ_H_* 1.44) was correlated with Val-2-C-2 (*δ_C_* 51.9) and Val-2-C-1 (*δ_C_* 171.1) in HMBC, while Val-2-H-2 (*δ_H_* 4.00) and Val-2-H-3 (*δ_H_* 1.44) showed correlations in the ^1^H-^1^H COSY spectrum, confirming the presence of other valine residues (Val-2). The isoleucine residue (Ile) was established by the correlations between Ile-H-5 (*δ_H_* 0.94) and Ile-C-3 (*δ_C_* 40.3) and Ile-C-4 (*δ_C_* 25.3), as well as between Ile-H-6 (*δ_H_* 1.02) and Ile-C-4 (*δ_C_* 25.3), Ile-C-3 (*δ_C_* 40.3), and Ile-C- 2 (*δ_C_* 60.9). Additionally, correlations between Ile-H-2 (*δ_H_* 3.90) and Ile-C-1 (*δ_C_* 169.2), Ile-C-3 (*δ_C_* 40.3), and Ile-C-4 (*δ_C_* 25.3) were noted, along with correlations among Ile-H-2/Ile-H-3/(Ile-H-6/)Ile-H-4/Ile-H-5 in the ^1^H-^1^H COSY spectrum. Combining the correlations among Leu-H-2/Leu-H-3/Leu-H-4/Leu-H-6/Leu-H-5 in the ^1^H-^1^H COSY spectrum, the leucine residue (Leu) was determined through the HMBC correlations between Leu-H-5 (*δ_H_* 0.95) and Leu-H-6 (*δ_H_* 0.96), and Leu-C-4 (*δ_C_* 25.6) and Leu-C-3 (*δ_C_* 45.1). Additionally, correlations between Leu-H-3 (*δ_H_* 1.72) and Leu-C-1 (*δ_C_* 171.0), Leu-C-2 (*δ_C_* 54.6), and Leu-C-4 (*δ_C_* 25.6) were observed. Additionally, Ile-H-2 (*δ_H_* 3.90) was correlated with Val-2-C-1 (*δ_C_* 171.1), and Leu-H-2 (*δ_H_* 3.93) was correlated with Ile-C-1 (*δ_C_* 169.2) in the HMBC spectrum (Figure 2). Compound **1** was identified as cyclo-(Val-Val-Ile-Leu) (Figure 2).

**Figure 3:**
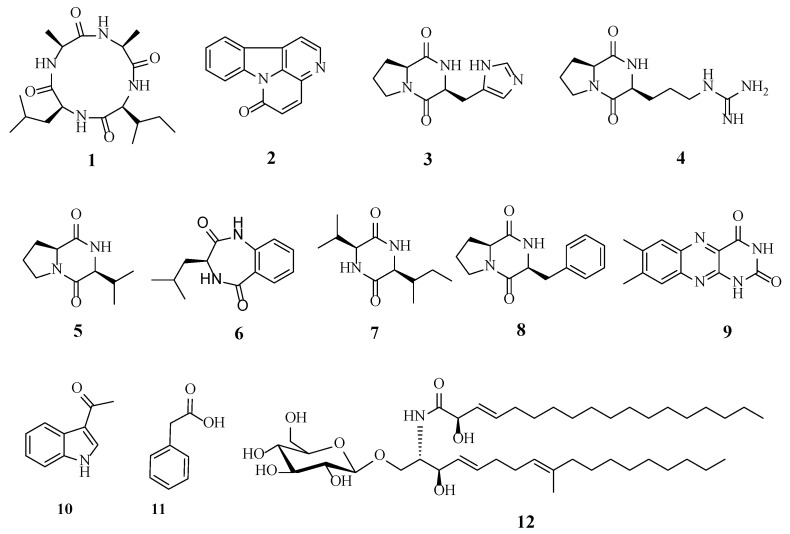
The metabolites isolated from *H. anguillulae* YMF1.01751.

Other known compounds were identified as canthin-6-one (**2**) [23], cyclo-(His-Pro) (**3**) [24], cyclo-(Arg-Pro) (**4**) [25], cyclo-(Pro-Val) (**5**) [26], 3-isobutyl-3,4-dihydro-1*H*-benzo[*e*][1,4]diazepine-2,5-dion (**6**) [27], cyclo-(Val-Ile) (**7**) [28], cyclo-(Pro-Phe) (**8**) [29], lumichrome (**9**) [30], 1-(1*H*-indol-3-yl)ethanone (**10**) [31], phenylacetic acid (**11**) [32], and cerebroside C (**12**) [33] based on the literature data. Their chemical structures are shown in Figure 3.

### 3.3. Nematicidal Activity of the Isolated Compounds Against M. incognita

**Figure 4:**
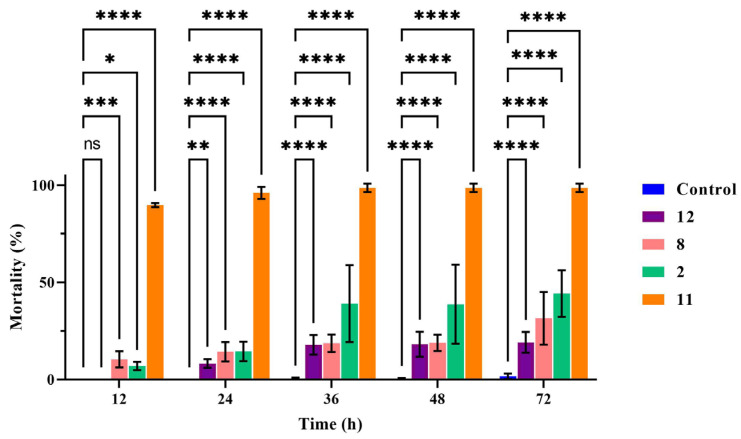
The nematicidal activity of compounds against *M. incognita*. In the same time period of data, two way-ANOVA statistical analysis indicates significant differences (* *p* < 0.05; ** *p* < 0.005; *** *p* < 0.003; **** *p* < 0.001) compared with the control.

The results showed that with increasing time up to 72 h, compounds **2**, **8**, **11**, and **12** exhibited enhanced nematicidal activity against *M. incognita* J2s at a concentration of 400 μg/mL. Compound **11** was the most active, and the nematode death rate reached 89.76% at 12 h and 96.05% at 24 h. The fatality rate of compound **2** was 44.26%, and that of compound **8** was 31.49% at 72 h at 400 μg/mL. In the further analysis, the nematicidal activity of compounds **2**, **8**, **11**, and **12** was shown to be significantly different to that of the blank control during all observation periods (Figure 4) by two way-ANOVA statistical analyses with GraphPad Prism 9.5.1. Furthermore, with avermectin, the nematode death rate reached 100% at 12 h at 100 μg/mL.

### 3.4. Chemotactic Activity of Compounds Towards M. incognita

**Figure 5:**
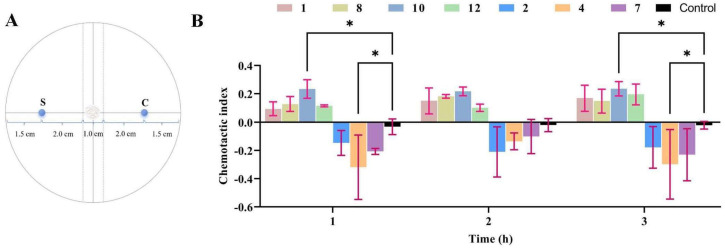
The chemotactic activity of compounds towards *M. incognita*. (**A**) Schematic diagram of chemotactic activity. (**B**) The chemotactic activity of compounds. In the same time period of data, two way-ANOVA statistical analysis indicates significant differences (* *p* < 0.05) compared with the control.

As described in the Materials and Methods Section, the sample and control were located in the S (experimental group) and C (blank group) areas, and root-knot nematode sampling areas were used in the center of the circle (Figure 5A). The chemotactic assay demonstrated that compound **10** exhibited chemotactic activity against the nematodes at 40 μg, with chemotactic indices of 0.234, 0.217, and 0.235 at 1, 2, and 3 h, respectively. Compounds **1**, **8**, and **12** showed weak attractive activity towards the nematodes, with the chemotactic index ranging from 0.1 to 0.2 at 1, 2, and 3 h (Figure 5B). In addition, *M. incognita* showed strong avoidance towards compound **4** at 40 μg, and the chemotactic indices were −0.298, −0.207, and −0.230, at 1, 2, and 3 h, respectively. Compounds **2** and **7** had a weak repulsion effect on root-knot nematodes, and the chemotactic index was between −0.1 and −0.2 (Figure 5B). Although some compounds exhibited certain chemotactic activity at the test concentrations in the observation periods, the results of further statistical analysis showed that only compounds **4** and **10** had significant differences at 1 and 3 h compared with the control (Figure 5B). A further chemotaxis experiment with compound **4** indicated that reducing compound **4** from 40 μg to 5 μg shifted its repulsion of root-knot nematodes, which gradually weakened.

## 4. Discussion

So far, there have only been two reports on the metabolites of the genus *Harposporium* [13,14]. In this study, specialized metabolites of *H. anguillulae* YMF1.01751 were discovered through culture condition screening, isolating twelve compounds from the cultures of *H. anguillulae* YMF1.01751 on CMA medium. All compounds were firstly obtained from the genus *Harposporium*. Their structure types were mainly aromatic compounds and cyclic peptides, and compound **1** was a new cyclic tetrapeptide. The nematicidal activity assay results showed that phenylacetic acid (**11**), cyclo-(Pro-Phe) (**8**), and canthin-6-one (**2**) possessed nematicidal activity against *M. incognita* J2s. Phenacetic acid (**11**) is a common and simple aromatic organic acid that can be produced by many microorganisms and has been reported to have nematicidal activity [34]. Compound **2** was mainly obtained from plants with antibacterial, antifungal, cytotoxic activities, and anti-inflammatory effects [35]. In addition, the chemotactic activity experiments showed that three compounds had attraction activity, while the other three had avoidance activity against *M. incognita* J2s. Then, two compounds, canthin-6-one (**2**) and (cyclo-(Pro-Phe) (**8**), showed nematicidal and chemotactic activity towards nematodes. These activities of the two compounds are reported for the first time. Therefore, exploring metabolites with various activities presents a promising research direction for developing targeted preparations for controlling root-knot nematodes. In addition, lumichrome (**9**) as a derivative of riboflavin, can stimulate the LasR bacterial QS receptor as a QS signal-mimic compound [36], and enhances root respiration in alfalfa and shoot growth [37]. Cerebroside C (**12**) was obtained from various fungi and plants as a potent elicitor and natural plant-growth regulator [38]. These results indicate that *H. anguillulae* can produce metabolites with diverse biological activities.

*H. anguillulae* is an important group of parasitic fungi and a major natural enemy of nematodes, primarily infecting bacterial nematodes (such as *Caenorhabditis elegans*) through endoparasitism. When *H. anguillulae* infects root-knot nematodes, which possess a narrow mouth needle, the nematode cannot swallow the spores. Instead, the fungus adheres to the nematode’s body surface through the spores, forming hyphae as the spores germinate. This process is accompanied by the secretion of extracellular enzymes and metabolites, which work with the hyphae to dissolve the nematode’s body wall and then infect it, resulting in its death [39]. The secondary metabolites of *H. anguillulae* YMF1.01751 under different culture conditions have different degrees of toxicity on root-knot nematodes, which suggests that the synthesis of the *H. anguillulae* YMF1.01751 metabolites is regulated by the environment, and that the production of these compounds has important physiological functions. An increasing number of studies indicate that metabolites are involved in the fungal infection of nematodes. Oligosporons and arthrosporols were identified from the nematode-trapping fungus *Arthrobotrys oligospora*; among them, oligosporon has toxic activity against intestinal parasitic nematodes [40], and arthrosporols participate in regulating the differentiation and formation of traps [41]. Interestingly, many fungi secrete metabolites to attract nematodes. *A. oligospora* produces the volatile compound methyl 3-methyl-2-butenoate, which strongly attracts nematodes [11], and 6-methylsalicylic acid, produced by *A. flagrans*, attracts nematodes and plays a spatiotemporal regulatory role in the formation of traps [12]. Subsequently, secondary metabolites may play an important role in the functions of fungal parasitic nematodes.

## 5. Conclusions

*H. anguillulae* YMF1.01751, a nematode endoparasitic fungus, has potential for the biological control of nematodes. In this study, its secondary metabolites were screened on different culture media, including CMA medium, leading to the isolation of 12 compounds and a new tetrapeptide. The biological activity screening results indicate that the strain can produce nematicidal and chemotactic active metabolites, which may play a role at different stages of fungal parasitism.

## Figures and Tables

**Table 1:** The NMR data of compound **1** in CD_3_OD.

Position	^1^H	^13^C	HMBC
Val-1-1	-	171.5, s	-
Val-1-2	4.03 (1H, q, *J* = 7.1 Hz)	51.7, d	20.88
Val-1-3	1.43 (3H, d, *J* = 7.1 Hz)	20.88, q	51.7, 171.5
Val-2-1	-	171.1, s	-
Val-2-2	4.00 (1H, q, *J* = 7.1 Hz)	51.9, d	20.92
Val-2-3	1.44 (3H, d, *J* = 7.1 Hz)	20.92, q	51.9, 171.1
Ile-1	-	169.2, s	-
Ile-2	3.90 (1H, d, *J* = 3.0 Hz)	60.9, d	15.6, 25.3, 40.3, 169.2, 171.1
Ile-3	1.95 (1H, m)	40.3, d	25.3, 60.9
Ile-4	1.24 (1H, m)	25.3, t	12.2, 40.3
	1.51 (1H, m)		12.2, 40.3
Ile-5	0.94 (3H, t, *J* = 7.4 Hz)	12.2, q	25.3, 40.3
Ile-6	1.02 (3H, d, *J* = 7.1 Hz)	15.6, q	25.3, 40.3, 60.9
Leu-1	-	171.0, s	-
Leu-2	3.93 (1H, dd, *J* = 4.6, 8.5 Hz)	54.6, d	25.6, 45.1, 171.0, 169.2
Leu-3	1.72 (1H, m)	45.1, t	23.5, 25.6, 54.6, 171.0
	1.63 (1H, m)		25.6, 54.6
Leu-4	1.84 (1H, m)	25.6, d	45.1, 22.1
Leu-5	0.95 (3H, d, *J* = 6.3 Hz)	22.1, q	25.6, 45.1
Leu-6	0.96 (3H, d, *J* = 6.2 Hz)	23.5, q	25.6, 45.1

## Data Availability

The original contributions presented in this study are included in the article/Supplementary Material. Further inquiries can be directed to the corresponding author.

## Supplementary Materials

The following supporting information can be downloaded at: https://www.mdpi.com/article/10.3390/microorganisms12122585/s1, Supplementary File: Materials and Methods. Figure S1. The nematicidal activity of the extracts from three media against *M. incognita*. In the same time period of data, two way-ANOVA statistical analysis indicates significant differences (*** *p* < 0.003; **** *p* < 0.001) compared with the control.
