# Supplementary material for: Bioactive Secondary Metabolites from Harposporium anguillulae Against Meloidogyne incognita
Source: Microorganisms. 2024 Dec 13;12(12):2585. doi: 10.3390/microorganisms12122585 (PMC11676538; doi:10.3390/microorganisms12122585)
Supplement: Supplementary file 1 [file microorganisms-12-02585-s001.zip › microorganisms-3335978-supplementary.pdf]

# Supplementary materials

# Bioactive secondary metabolites from *Harposporium anguillulae* against *Meloidogyne incognita*

Dong Li[1#], · Ling-Feng Bao[2#], · Hong-Mei Lei[1], · Guang-Ke Zhang[1], · Guo-Hong Li[1] and Pei-Ji Zhao[1*]

[1] State key Laboratory for Conservation and Utilization of Bio-Resources in Yunnan, School of Life Sciences, Yunnan University, Kunming 650091, China

**[2]** Institute of Tropical Eco-Agricltural Sciences of Yunnan Academy of Agricultural Sciences, Kunming 650091, China

# These authors have contributed equally to this work.
*Correspondence: pjzhao@ynu.edu.cn (P.Z.)

**Materials and Methods**

*General Experimental Instruments*

Optical rotations were measured with a Jasco DIP-370 digital polarimeter. The nuclear magnetic resonance (NMR) spectra were recorded on Avance III-600 spectrometers with tetramethylsilane (TMS) as an internal standard. The electrospray ionization mass spectra (ESI-MS) and high resolution electrospray ionization mass spectra (HR-ESI-MS) were recorded on a Thermo high resolution Q Exactive mass spectrometer (Thermo, Bremen, Germany). Column chromatography was performed on silica gel (200-300 mesh, Qingdao Marine Chemical Inc., Qingdao, China), Sephadex LH-20 (Amersham Pharmacia) and Silica gel 60 RP-18 (40-63 μm, Merck). Precoated silica gel GF254 plates (Qingdao Marine Chemical Inc., Qingdao, China) were used for thin-layer chromatography (TLC). Fractions were monitored by TLC and visualized by heating plates sprayed with 5% $H_2SO_4$ in EtOH. Semipreparative HPLC was performed on an LC3000 (Beijing Chuangxintongheng Science & Technology Co., Ltd, Beijing, China). The organic solvents in the experiment were all industrial grade and used after reevaporation and recovery. Deuterated NMR solvents ($CD_3OD$, $CDCl_3$, $C_5D_5N$, and DMSO-$d_6$ ) were purchased from Cambridge Isotope Laboratories.

*Extraction and Isolation of Metabolites*

The 30 L CMA medium was prepared. Each plate was inoculated with 500 μL conidial suspension of *H. anguillulae* YMF1.01751 and cultured at 28°C for 21 days. After cultivation, the cultures were extracts by organic solvent (EtOAc/MeOH/AcOH = 80:15:5, *v*/*v*/*v*) at least 3 times, and the extract was concentrated by rotary evaporation. Finally, organic solvent (chloroform-methanol, 1:1) was repeatedly used to dissolve to get the extract (105 g).

The extract (105 g) was placed on a reversed-phase silica gel (60×6 cm, RP-18) column eluting with $H_2O$/MeOH mixtures (100:0, 90:10, 70:30, 50:50, 30:70, 10:90, and 0:100) to obtain 7 fractions (A1-A7). A1 (25.937 g) was separated by Sephadex LH-20 (150×3 cm, chloroform–methanol, 1:1) to obtain 3 fractions (A1-1 to A1-3).

Fraction A1-3 (1.39 g) was separated by a silica gel column (60×2 cm) eluting with petroleum ether–ethyl acetate (50:1→7:3) and further purified by a silica gel column (40×1 cm, chloroform–acetone, 30:1→7:3) to obtain **11** (2.3 mg). Fraction A2 (3.51 g) was placed on a silica gel column (60×2 cm) eluting with petroleum ether–acetone to obtain 3 fractions (A2-1 to A2-3). Fraction A2-1 (1.11 g) was purified on a silica gel column (60×2 cm) eluting with chloroform–acetone (50:1→7:3) and further isolated by Sephadex LH-20 (100×1 cm, acetone) to obtain **10** (2 mg). Fraction A3 (1.97 g) was separated on a RP-18 column (60×2 cm) eluting with $H_2O$–MeOH mixtures (100:0, 90:10, 70:30, 50:50, 30:70, 10:90, and 0:100) to obtain 5 fractions (A3-1 to A3-5). Fraction A3-2 (26.7 mg) was separated by Sephadex LH-20 (100×1 cm, methanol) to obtain 4 fractions (A3-2-1 to A3-2-4). Fraction A3-2-1 (12 mg) was subjected on a silica gel column (40×1 cm) eluting with petroleum ether–acetone (50:1→7:3) to obtain **8** (7.2 mg). Fraction A3-2-2 (41.5 mg) was purified by a silica gel column (40×1 cm) eluting with chloroform–methanol (40:1→10:1) to obtain **12** (2.5 mg). Fraction A3-2-3 (14.5 mg) was separated by Sephadex LH-20 (100×1 cm, acetone) and further purified on a silica gel column eluting with chloroform–methanol (20:1) to obtain **6** (1.9 mg). Fraction A3-2-4 (45.4 mg) was purified by a silica gel column (40×1 cm) eluting with chloroform–methanol (10:1) to obtain **9** (20 mg). Fraction A4 (32.86 mg) was isolated with semipreparative gradient HPLC with the mobile phase MeOH–$H_2O$ (30:70→100:0) for 30 min at 254 nm and then purified by Sephadex LH-20 (methanol) to obtain **2** (2.1 mg). Fraction A5 (2.1 g) was isolated by a silica gel column (60×2 cm) eluting with petroleum ether–ethyl acetate (10:1→7:3) and then chloroform–methanol (20:1→8:2) to obtain 4 fractions (A5-1 to A5-4). Fraction A5-1 (1.3 g) was placed on a silica gel column (60×2 cm) eluting with petroleum ether–acetone (10:1→7:3) and then chloroform→methanol (20:1→8:2) to obtain 4 fractions (A5-1-1 to A5-1-4). A5-1-4 (60 mg) was subjected on semipreparative gradient HPLC with the mobile phase MeOH–$H_2O$ (30:70→100:0) for 30 min at 254 nm and then purified by a silica gel column eluting with chloroform–methanol (40:1) to obtain **5** (5 mg). Fraction A5-2 (111.2 mg) was subjected on Sephadex LH-20 (100×1 cm, chloroform–methanol, 1:1) and then

purified by a silica gel column eluting with chloroform–methanol (40:1) to obtain **3** (5 mg). Fraction A5-3 (17 mg) was isolated with semipreparative gradient HPLC (MeOH–$H_2O$, 20:80→100:0) for 25 min at 254 nm and then purified by a silica gel column (40×1 cm) eluting with chloroform–acetone (10:1) to obtain **1** (2.8 mg). Fraction A5-4 (85 mg) was subjected on Sephadex LH-20 (100×1 cm, methanol) and then purified by a silica gel column (40×1 cm) eluting with ethyl acetate–methanol (4:1) to obtain **4** (3.3 mg). Fraction A7 (1.1 g) was separated on a silica gel column (60×2 cm) eluting with chloroform-methanol (40:1→1:1) to obtain 3 fractions (A7-1 to A7-3). Fraction A7-2 (84.6 mg) was subjected to Sephadex LH-20 (100×1 cm, chloroform–methanol, 1:1) and then purified by recrystallization in petroleum ether and ethyl acetate to obtain **7** (2.5 mg).

*Spectral Data of Known Compounds*

Canthin-6-one (**2**): Yellowish solid; $C_{14}H_8N_2O$; ESI-MS *m/z*: 221 $[M + H]^+$; $^1$H-NMR ($CD_3OD$, 600 MHz) δ: 6.63 (1H, d, *J* = 7.8 Hz, H-15), 7.56 (1H, t, *J* = 7.2 Hz, H-10), 7.78 (1H, t, *J* = 7.8 Hz, H-11), 8.03 (1H, d, *J* = 8.4 Hz, H-6), 8.31 (1H, d, *J* = 7.8 Hz, H-12), 8.38 (1H, d, *J* = 4.8 Hz, H-9), 8.83 (1H, d, *J* = 7.8 Hz, H-16), 8.97 (1H, d, *J* = 4.8 Hz, H-5); $^{13}$C-NMR ($CD_3OD$, 150 MHz) δ: 113.0 (C-7), 117.2 (C-12), 120.8 (C-11), 125.2 (C-9), 125.7 (C-10), 126.5 (C-6), 132.6 (C-2), 135.5 (C-8), 136.0 (C-15), 136.3 (C-13), 139.0 (C-5), 141.1 (C-16), 148.5 (C-3), 181.1 (C-14).

Cyclo-(His-Pro) (**3**): Yellowish solid; $C_{11}H_{14}N_4O_2$; ESI-MS *m/z*: 235 $[M + H]^+$; $^1$H-NMR ($CD_3OD$, 600 MHz) δ: 7.64 (1H, s, His-H-6), 6.93 (1H, s, His-H-5), 4.37~4.39 (1H, m, His-H-1), 4.20~4.23 (1H, m, Pro-H-2), 3.49~3.56 (2H, m, Pro-H-5), 3.29~3.01 (2H, m, His-H-2), 2.25~2.27 (2H, m, Pro-H-3), 1.82~1.98 (2H, m, Pro-H-4); $^{13}$C-NMR ($CD_3OD$, 150 MHz) δ: 172.0 (Pro-C-1), 167.5 (His-C-1), 136.4 (His-C-4), 136.3 (His-C-6), 118.8 (His-C-5), 60.3 (Pro-C-2), 56.5 (His-C-2), 46.4 (Pro-C-5), 29.3 (Pro-C-3), 28.5 (His-C-3), 23.4 (Pro-C-4).

Cyclo-(Arg-Pro) (**4**): Yellowish solid; $C_{11}H_{18}N_5O_2$; ESI-MS *m/z*: 254 $[M + H]^+$; $^1$H-NMR ($CD_3OD$, 600 MHz) δ: 4.24 (1H, m, Pro-H-2), 4.22 (1H, m, Arg-H-2), 3.51 (2H, t, *J* = 9.0 Hz, Pro-H-5), 3.19 (2H, t, *J* = 7.2 Hz, Arg-H-5), 2.31 (2H, brt, Pro-H-3),

1.88~2.00 (4H, m, Pro-H-4/Arg-H-3), 1.63~1.69 (2H, m, Arg-H-4); $^{13}$C-NMR ($CD_3OD$, 150 MHz) δ: 172.6 (Arg-C-1), 167.9 (Pro-C-1), 158.7 (Arg-C-6), 60.4 (Pro-C-2), 55.9 (Arg-C-2), 46.4 (Pro-C-5), 42.3 (Arg-C-5), 29.3 (Pro-C-3), 27.5 (Arg-C-3), 25.1 (Pro-C-4), 23.5 (Arg-C-4).

Cyclo-(Pro-Val) (**5**): White solid; $C_{10}H_{16}N_2O_2$; ESI-MS *m/z*: 197 $[M + H]^+$; $^1$H-NMR ($CDCl_3$, 600 MHz) δ: 6.21 (1H, s, NH), 4.06 (1H, dd, *J* = 7.8, 15.6 Hz, Pro-H-2), 3.93 (1H, m, Val-H-2), 3.57 (2H, m, Pro-H-5), 2.62 (1H, m, Val-H-3), 2.36 (1H, m, Pro-H-3a), 1.80~2.05 (3H, m, Pro-H-4/Pro-H-3b), 1.06 (3H, d, *J* = 7.2 Hz, Val-H-4), 0.89 (3H, d, *J* = 6.6 Hz, Val-H-5); $^{13}$C-NMR ($CDCl_3$, 150 MHz) δ: 170.1 (Val-CO), 164.9 (Pro-CO), 60.4 (Val-C-2), 68.8 (Pro-C-2), 45.1 (Pro-C-5), 28.4 (Val-C-3), 28.3 (Pro-C-3), 22.3 (Pro-C-4), 19.2 (Val-C-4), 16.0 (Val-C-5).

3-Isobutyl-3,4-dihydro-1*H*-benzo[*e*][1,4]diazepine-2,5-dion (**6**): White solid; $C_{13}H_{16}N_2O_2$; ESI-MS *m/z*: 233 $[M + H]^+$; $^1$H-NMR ($CDCl_3$, 600 MHz) δ: 0.89 (3H, d, *J* = 6.6 Hz, H-5/H-6), 0.96 (3H, d, *J* = 6.6 Hz, H-5/H-6), 1.25 (m, 1H, H-4), 1.56 (m, 1H, H-3a), 1.64 (m, 1H, H-3b), 3.80 (1H, m, H-2), 6.98-7.53 (4H, m, Ar-H); $^{13}$C-NMR ($CDCl_3$, 150 MHz) δ: 21.8 (C-5/C-6), 22.9 (C-5/C-6), 24.4 (C-4), 37.3 (C-3), 50.4 (C-2), 120.8 (Ar-C-4), 125.4 (Ar-C-6), 125.6 (Ar-C-2), 131.5 (Ar-C-7), 133.2 (Ar-C-5), 135.5 (Ar-C-3), 168.3 (CO), 171.4 (CO).

Cyclo-(Val-Ile) (**7**): White solid; $C_{11}H_{20}N_2O_2$; ESI-MS *m/z*: 213 $[M + H]^+$; $^1$H-NMR ($C_5D_5N$, 600 MHz) δ: 8.85 (2H, brs, NH), 4.27 (1H, brs, Ile-H-2), 4.14 (1H, brs, Val-H-2), 2.51 (1H, m, Val-H-3), 2.15 (1H, m, Ile-H-3), 1.44 (1H, m, Ile-H-4a), 1.18 (1H, m, Ile-H-4b), 1.11 (3H, d, *J* = 6.0 Hz, Val-H-4), 1.10 (3H, d, *J* = 6.0 Hz, Val-H-5), 0.94 (3H, d, *J* = 6.0 Hz, Ile-H-6), 0.91 (3H, t, *J* = 6.0 Hz, Ile-H-5); $^{13}$C-NMR ($C_5D_5N$, 150 MHz) δ: 168.4 (Val-C-1), 168.2 (Ile-C-1), 60.5 (Val-C-2), 60.0 (Ile-C-2), 39.0 (Ile-C-3), 32.0 (Val-C-3), 23.3 (Ile-C-4), 19.2 (Val-C-4), 17.5 (Val-C-5), 15.6 (Ile-C-5), 12.1 (Ile-C-6).

Cyclo-(Pro-Phe) (**8**): White solid; $C_{14}H_{16}N_2O_2$; ESI-MS *m/z*: 245 $[M + H]^+$; $^1$H-NMR ($CDCl_3$, 600 MHz) δ: 7.22~7.36 (5H, m, Phe-H-Ph), 5.68 (1H, bs, Phe-NH), 4.27 (1H, m, Phe-H-2), 4.07 (1H, m, Pro-H-2), 3.54~3.67 (4H, m, Pro-H-5/Phe-H-3), 2.78 (1H, m, Pro-H-3a), 2.32 (1H, m, Pro-H-3b), 1.89~2.04 (2H, m, Pro-H-4);

$^{13}C$-NMR ($CDCl_3$, 150 MHz) δ: 169.4 (Pro-CO), 165.0 (Phe-CO), 136.0 (Phe-C-4), 129.1 (Phe-C-5, Phe-C-9), 129.2 (Phe-C-6, Phe-C-8), 127.5 (Phe-C-7), 59.1 (Pro-C-2), 56.1 (Phe-C-2), 45.4 (Pro-H-5), 36.7 (Phe-C-3), 28.3 (Pro-C-3), 22.5 (Pro-C-4).

Lumichrome (**9**): Yellowish powder; $C_{12}H_{10}N_4O_2$; ESI-MS *m/z*: 243 $[M + H]^+$; $^1H$-NMR (DMSO-$d_6$, 600 MHz) δ: 7.90 (1H, s, H-6), 7.70 (1H, s, H-9), 2.48 (3H, s, H-12), 2.46 (3H, s, H-11); $^{13}C$-NMR (DMSO-$d_6$, 150 MHz) δ: 160.7 (C-4), 150.1 (C-2), 146.5 (C-10a), 144.8 (C-7), 141.7 (C-9a), 139.0 (C-8), 138.4 (C-5a), 130.4 (C-4a), 128.7 (C-6), 125.9 (C-9), 20.3 (C-12), 19.6 (C-11).

1-(1*H*-Indol-3-yl)ethanone (**10**): White solid; $C_{10}H_9NO$; ESI-MS *m/z*: 160 $[M + H]^+$; $^1H$-NMR ($CDCl_3$, 600 MHz) δ: 10.30 (1H, s, -NH), 8.55 (1H, s, H-1′), 8.16 (2H, m, H-4′/H-7′), 7.59~7.63 (2H, m, H-5′/H-6′), 2.98 (3H, s, H-3); $^{13}C$-NMR ($CDCl_3$, 150 MHz) δ: 25.9 (C-1), 112.0 (C-7′), 119.1 (C-2′), 120.6 (C-5′), 120.7 (C-4′), 121.8 (C-6′), 129.3 (C-3′), 131.6 (C-8′), 138.1 (C-1′), 203.3 (C-2).

Phenylacetic acid (**11**): White solid; $C_8H_8O_2$; ESI-MS *m/z*: 137 $[M + H]^+$; $^1H$-NMR ($CDCl_3$, 600 MHz) δ: 3.64 (2H, s, H-2), 7.26~7.28 (5H, m, H-2/H-3/H-4/H-5/H-6); $^{13}C$-NMR ($CDCl_3$, 150 MHz) δ: 177.2 (C-8), 133.5 (C-1), 129.4 (C-2/C-6), 128.6 (C-3/C-5), 127.3 (C-4), 41.2 (C-7).

Cerebroside C (**12**): White solid; $C_{43}H_{79}NO_9$; ESI-MS *m/z*: 776 $[M + Na]^+$; $^1H$-NMR ($CD_3OD$, 600 MHz) δ: 0.89 (6H, t, *J* = 6.8 Hz, H-18/H-18'), 1.30 (34H, m, H-12~H-17, H-7'~H-17'), 1.38~1.41 (4H, m, H-11/H-6'), 1.59 (3H, s, H-19), 1.97 (2H, m, H-10), 2.02 (2H, m, H-5'), 2.02 (2H, m, H-6), 2.07 (2H, m, H-7), 3.20 (1H, dd, *J* = 10.8, 6.6 Hz, H-2"), 3.29 (1H, m, H-4"/5"), 3.30 (1H, m, H-4"/5"), 3.35 (1H, dd, *J* = 7.8 Hz, H-3"), 3.68 (1H, dd, *J* = 12.0 Hz, H-6"), 3.71 (1H, dd, *J* = 10.8, 3.6 Hz, H-1), 3.87 (1H, d, *J* = 12.0 Hz, H-6"), 3.97 (1H, dt, *J* = 6.0, 3.6 Hz, H-2), 4.10 (1H, dd, *J* = 7.8, 3.0 Hz, H-1), 4.13 (1H, dd, *J* = 10.2, 5.4 Hz, H-3), 4.27 (1H, d, *J* = 7.8 Hz, H-1"), 4.43 (1H, d, *J* = 6.0 Hz, H-2'), 5.14 (1H, t, *J* = 1.2 Hz, H-8), 5.46 (1H, dd, *J* = 15.6, 7.8 Hz, H-4), 5.47 (1H, dd, *J* = 13.8, 6.0 Hz, H-3'), 5.48 (1H, s, H-5), 5.49 (1H, s, H-4'); $^{13}C$-NMR ($CD_3OD$, 150 MHz) δ: 175.4 (C-1'), 136.7 (C-9), 134.7 (C-4'), 134.5 (C-5), 131.1 (C-4), 129.0 (C-3'), 124.8 (C-8), 104.7 (C-1"), 78.0 (C-5"), 77.9 (C-3"), 75.0 (C-2"), 74.1 (C-2'), 73.1 (C-3), 71.6 (C-4"), 69.7 (C-1), 62.7 (C-6"), 54.6 (C-2),

40.8 (C-10), 33.8 (C-6), 33.5 (C-5'), 33.1 (C-16'), 33.1 (C-16), 30.2~30.8 (C-12~C-15), 30.2~30.8 (C-6'~C-15'), 29.1 (C-11), 28.8 (C-7), 23.8 (C-17'), 23.9 (C-17), 16.2 (C-19), 14.5 (C-18'), 14.5 (C-18).

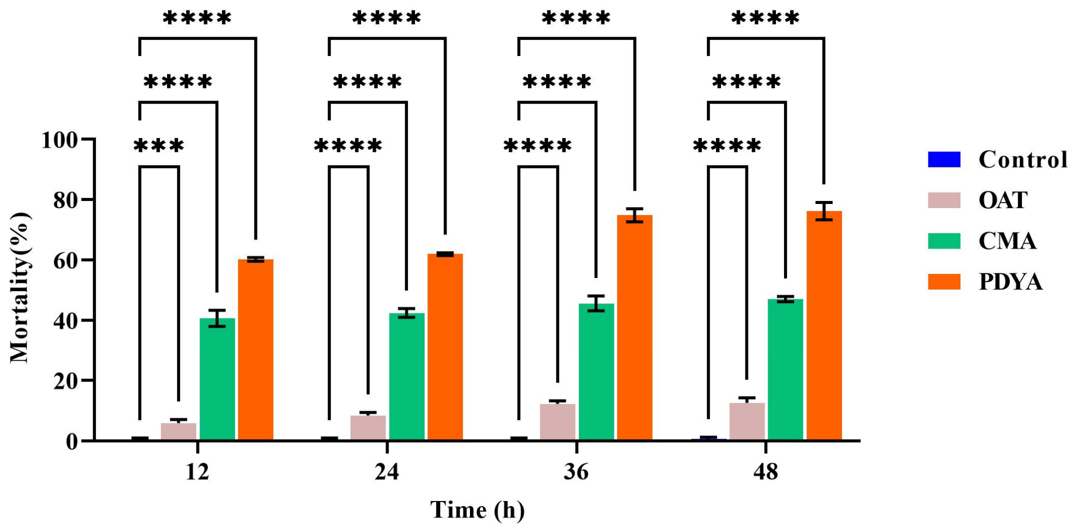


**Figure S1.** The nematicidal activity of the extracts from three media against *M. incognita*. In the same time period of data, two way-ANOVA statistical analysis indicates significant differences (***$P$<0.003; ****$P$<0.001) compared with the control.
